# Origin of the MRI Contrast in Natural and Hydrogel Formulation of Pineapple Juice

**DOI:** 10.1155/2021/6666018

**Published:** 2021-01-05

**Authors:** Domenico Rizzo, Enrico Ravera, Marco Fragai, Giacomo Parigi, Claudio Luchinat

**Affiliations:** ^1^Magnetic Resonance Center (CERM), Department of Chemistry, University of Florence, Via Sacconi 6, Sesto Fiorentino, Florence, Italy; ^2^Consorzio Interuniversitario Risonanze Magnetiche di Metalloproteine (CIRMMP), Sesto Fiorentino, Florence, Italy

## Abstract

Magnetic resonance imaging (MRI) often requires contrast agents to improve the visualization in some tissues and organs, including the gastrointestinal tract. In this latter case, instead of intravascular administration, oral agents can be used. Natural oral contrast agents, such as fruit juice, have the advantages of better taste, tolerability, and lower price with respect to the artificial agents. We have characterized the relaxometry profiles of pineapple juice in order to understand the origin of the increase in relaxation rates (and thus of the MRI contrast) in reference to its content of manganese ions. Furthermore, we have characterized the relaxometry profiles of pineapple juice in the presence of alginate in different amounts; the interaction of the manganese ions with alginate slows down their reorientation time to some extent, with a subsequent increase in the relaxation rates. The relaxometry profiles were also compared with those of manganese(II) solutions in 50 mmol/dm^3^ sodium acetate solution (same pH of pineapple juice), which revealed sizable differences, mostly in the number of water molecules coordinated to the metal ion, their lifetimes, and in the constant of the Fermi-contact interaction. Finally, the fit of the transverse relaxivity shows that the increased viscosity in the hydrogel formulations can improve significantly the negative contrast of pineapple juice at the magnetic fields relevant for clinical MRI.

## 1. Introduction

Fruit juice rich in manganese ions is increasingly used in clinical MRI as oral contrast agent for imaging of the gastrointestinal tract [1]. Magnetic resonance cholangiopancreatography images are in fact frequently degraded by the high signal due to the fluid collecting in stomach and duodenum. Oral contrast agents can greatly improve the visualization of both biliary tree and pancreatic ducts, facilitating the evaluation of distal ducts, and of the stomach and small bowel. Oral manganese ions were also shown to provide good MRI contrast for the imaging of peripheral nerves involved in pain perception, thanks to the ability of Mn^2+^ to enter neurons via calcium channels [2].

The ideal oral agent must be nontoxic, increase the contrast homogeneously even when diluted through the gastrointestinal tract, and have good digestive acceptance, absence of collateral effects, minimum peristaltic stimulation, and an accessible price [3]. Several natural substances have been tried because of their content of Mn^2+^ ions [4–9]. Natural commercially available manganese-rich fruits include pineapple, blueberry, raspberry, blackberry, medlar, plum, and the Amazonian fruit pulp from Euterpe oleracea (popularly named Açaí) [10–12]. Pineapple juice is the most promising natural food for higher content of Mn^2+^ ions [13, 14], providing image contrast of a degree similar to commercially available negative contrast agents [6]. Since Mn^2+^ ions can largely increase both the longitudinal and the transverse relaxation rate of water protons, it can be used as both T1 and T2 contrast agent. For instance, as T2 agent, it was shown to suppress the signal from bowel fluid in pediatric magnetic resonance cholangiopancreatography, while as T1 agent, it can delineate the gut [13].

Pineapple juice has been shown effective as an oral contrast agent in magnetic resonance images, and, more recently, concentrated juices added with hydrogels [15] have been proposed to further enhance the contrast. However, so far, no detailed studies were performed on natural juices or on their derivatives to characterize the relaxation properties in detail. Field-dependent relaxometry [16, 17], which consists in the measurement of the nuclear relaxation rates as a function of the applied magnetic field, is a technique of choice in this respect. In fact, it provides access to a number of physicochemical parameters upon which the relaxation rates depend [18–20]. In the presence of paramagnetic systems, they are structural parameters, such as the number and distance of water molecules coordinated to the paramagnetic metal ions, and motional parameters, such as the reorientation time of the system, the lifetimes of the water molecules coordinated to the metal, the electron relaxation parameters, and the unpaired electron spin density delocalized onto the water protons [17]. From the values of these parameters, it is possible to infer on the occurring interactions of the paramagnetic metal with its environment and to monitor how they change upon changing the experimental conditions. Furthermore, the analysis of the relaxometry profiles permits to evaluate the different contributions to relaxation arising from the modulation of different types of metal-proton interactions and from the different motional regimes.

In this study, we characterize the relaxometry profile of pineapple juice in order to describe the relevance of the different parameters determining the experimental rates. We also analyze the effect of alginate added to pineapple juice as a natural food able to slower the dynamics of the paramagnetic ions present in the juice and thus to increase the nuclear relaxation rates. As manganese ions represent the major actors driving the relaxation properties of pineapple juice, the relaxation rates of solutions of manganese ions in conditions similar to those of the juice are also investigated, in order to evaluate the effects that other components than these ions have on the relaxation rates of the juice.

## 2. Materials and Methods

### 2.1. Sample Preparation

Samples were prepared by dissolving sodium alginate (Sigma-Aldrich, A1112) in pineapple juice (Coop, 100%, pineapple juice) or in a simulated pineapple juice to obtain hydrogels at 5% and 15% (w/w) of polysaccharide. The simulated pineapple juice containing the same concentration of inorganic salts (4 mmol/dm^3^ Ca^2+^; 6 mmol/dm^3^ Mg^2+^; 0.437 mmol/dm^3^ Mn^2+^) and the same pH (3.6) of the natural product were prepared by dissolving stock solutions of MgCl_2_, CaCl_2_, and MnCl_2_ in 50 mmol/dm^3^ of sodium acetate. Sodium acetate solution was prepared by dissolving pure acetic acid in water and then by adding a concentrated solution of sodium hydroxide until pH 3.6 is reached. Water was then added to match the desired concentration. The hydrogels were homogenized by several steps of sonication.

### 2.2. ^1^H NMRD Measurements

Water relaxation profiles were acquired with a Stelar Spinmaster FFC2000-1T relaxometer by measuring the water proton relaxation rates as a function of the applied magnetic field (0.01–40 MHz proton Larmor frequency). The relaxation measurements, obtained from the fit of the magnetization decay/recovery curves against a monoexponential function, were affected by an error of about ±1%. The relaxivity profiles were obtained by normalization of the measured relaxation data, subtraction of the diamagnetic relaxation rate contribution, to the Mn^2+^ concentration (in mmol/dm^3^). The measurements were performed at 25 and 37°C.

### 2.3. High-Field NMR Measurements

*R*_1_ and *R*_2_ at high field were measured on a Bruker Avance III spectrometer operating at 400 MHz ^1^H Larmor frequency (9.4 T) to mitigate the effects of relaxation, using a 5 mm BBO probehead. To mitigate the effect of radiation damping, the samples were put in a single capillary coaxial to the 5 mm tube, and the probehead was detuned by 1 MHz.

### 2.4. Model Used to Analyze the Data

The experimental water proton relaxation rates *R*_1_=*R*_1dia_+*R*_1*p*_ and *R*_2_=*R*_2dia_+*R*_2*p*_ are composed of diamagnetic and paramagnetic contributions. The paramagnetic contributions are related to the presence of paramagnetic metal ions in solution, which increase the relaxation rates of the water protons coordinated to the metal ions through hyperfine coupling. These paramagnetic relaxation enhancements (*R*_1*M*_ and *R*_2*M*_) are then transferred to bulk water protons through chemical exchange so that

(1) $$\begin{array}{c} R_{1p}=f_{M}{\left(R_{1M}^{-1}+\tau_{M}\right)}^{-1}+R_{1\text{out}}, \end{array}$$

(2) $$\begin{array}{c} R_{2p}=\frac{f_{M}}{\tau_{M}}\frac{R_{2M}^{2}+R_{2M}\tau_{M}^{-1}+{\left(\Delta \omega_{M}\right)}^{2}}{{\left(R_{2M}+\tau_{M}^{-1}\right)}^{2}+{\left(\Delta \omega_{M}\right)}^{2}}+R_{2\text{out}}≅f_{M}{\left(R_{2M}^{-1}+\tau_{M}\right)}^{-1}+R_{2\text{out}}, \end{array}$$

being the difference in chemical shift Δ*ω*_*M*_ between the paramagnetic and the diamagnetic species much smaller than *R*_2*M*_, where

(3) $$\begin{array}{c} f_{M}=\frac{q\left[{\text{Mn}}^{2+}\right]}{55.5}. \end{array}$$

The lifetime *τ*_*M*_ is the inverse of the exchange rate, *q* is the number of water molecules coordinated to the metal ion, and *R*_1out_ and *R*_2out_ indicate the paramagnetic relaxation enhancements due to longitudinal and transverse translational diffusion (also called outer sphere relaxation), i.e., due to the dipole-dipole interaction between the paramagnetic metal ion and the water molecules freely diffusing around it up to a distance of closest approach *d*.

*R*_1*M*_ and *R*_2*M*_ can be described by the Solomon–Bloembergen–Morgan (SBM) model [21, 22] and are composed of the Fermi-contact and dipolar contributions:

(4) $$\begin{array}{c} R_{1M}=\frac{2S\left(S+1\right)}{3}{\left(\frac{A^{\text{FC}}}{\hbar }\right)}^{2}\left[\frac{\tau_{\text{FC}}}{1+\omega_{S}^{2}\tau_{\text{FC}}^{2}}\right]+\frac{2}{15}{\left(\frac{\mu_{0}}{4\pi }\frac{\gamma_{I}g_{\text{iso}}\mu_{B}}{r^{3}}\right)}^{2}S\left(S+1\right)\left\{S_{LS}^{2}\left[\frac{7\tau_{c}}{1+\omega_{s}^{2}\tau_{c}^{2}}+\frac{3\tau_{c}}{1+\omega_{I}^{2}\tau_{c}^{2}}\right]+\left(1-S_{LS}^{2}\right)\left[\frac{7\tau_{f}}{1+\omega_{s}^{2}\tau_{f}^{2}}+\frac{3\tau_{f}}{1+\omega_{I}^{2}\tau_{f}^{2}}\right]\right\}, \end{array}$$

(5) $$\begin{array}{c} R_{2M}=\frac{S\left(S+1\right)}{3}{\left(\frac{A^{\text{FC}}}{h}\right)}^{2}\left[\tau^{\text{FC}}+\frac{\tau_{\text{FC}}}{1+\omega_{S}^{2}\tau_{\text{FC}}^{2}}\right]+\frac{1}{15}{\left(\frac{\mu_{0}}{4\pi }\frac{\gamma_{I}g_{\mathrm{iso}}\mu_{B}}{r^{3}}\right)}^{2}S\left(S+1\right)\left\{S_{LS}^{2}\left[4\tau_{c}+\frac{13\tau_{c}}{1+\omega_{s}^{2}\tau_{c}^{2}}+\frac{3\tau_{c}}{1+\omega_{I}^{2}\tau_{c}^{2}}\right]+\left(1-S_{LS}^{2}\right)\left[4\tau_{f}+\frac{13\tau_{f}}{1+\omega_{s}^{2}\tau_{f}^{2}}+\frac{3\tau_{f}}{1+\omega_{I}^{2}\tau_{f}^{2}}\right]\right\}, \end{array}$$

where (*A*^FC^/*h*) is the contact coupling constant, *r* is the distance between metal ion and coordinated protons, *S* is the electron spin quantum number (5/2 in the case of Mn^2+^), *τ*_FC_ is the correlation time for the Fermi-contact interaction:

(6) $$\begin{array}{c} \tau_{\text{FC}}^{-1}=\tau_{e}^{-1}+\tau_{M}^{-1}, \end{array}$$

and *τ*_*c*_ is the correlation time for the dipole-dipole interaction, given by

(7) $$\begin{array}{c} \tau_{c}^{-1}=\tau_{r}^{-1}+\tau_{e}^{-1}+\tau_{M}^{-1}, \end{array}$$

where *τ*_*r*_ is the tumbling time of the paramagnetic system and *τ*_*e*_ is the electron relaxation time, and

(8) $$\begin{array}{c} \tau_{e}^{-1}=\frac{2\Delta_{t}^{2}}{50}\left[4S\left(S+1\right)-3\right]\left[\frac{\tau_{v}}{1+\omega_{S}^{2}\tau_{v}^{2}}+\frac{4\tau_{v}}{1+4\omega_{S}^{2}\tau_{v}^{2}}\right], \end{array}$$

described in the pseudorotation model by the parameters Δ_*t*_ and *τ*_*v*_, which correspond to the transient zero-field splitting and the correlation time for electron relaxation, respectively. Using the Lipari and Szabo model-free approach [23], in equations (4) and (5), a squared order parameter *S*_*LS*_^2^ is also introduced to account for contributions arising in the presence of nonrigid molecular reorientations. In this case, in fact, the dipole-dipole interaction is partially modulated with a further correlation time (*τ*_*f*_) which is also determined by the internal faster mobility, with correlation time *τ*_*ι*_:

(9) $$\begin{array}{c} \tau_{f}^{-1}=\tau_{r}^{-1}+\tau_{e}^{-1}+\tau_{M}^{-1}+\tau_{\iota }^{-1}. \end{array}$$

Other symbols in equations (4) and (5) have the usual meaning [17]. The outer sphere relaxation can be described using the Freed model [24]:

(10) $$\begin{array}{c} R_{1}^{\text{out}}=\frac{32\pi }{405}{\left(\frac{\mu_{0}}{4\pi }\right)}^{2}\frac{1000N_{A}\left[{\text{Mn}}^{2+}\right]{\left(\gamma_{I}\mu_{B}g_{e}\right)}^{2}S\left(S+1\right)}{d\;D}\left[7J^{F}\left(\omega_{S},\tau_{D}\right)+3J^{F}\left(\omega_{I},\tau_{D}\right)\right], \end{array}$$

(11) $$\begin{array}{c} R_{2}^{\text{out}}=\frac{16\pi }{405}{\left(\frac{\mu_{0}}{4\pi }\right)}^{2}\frac{1000N_{A}\left[{\text{Mn}}^{2+}\right]{\left(\gamma_{I}\mu_{B}g_{e}\right)}^{2}S\left(S+1\right)}{d\;D}\left[4+13J^{F}\left(\omega_{S},\tau_{D}\right)+3J^{F}\left(\omega_{I},\tau_{D}\right)\right], \end{array}$$

where

(12) $$\begin{array}{c} J^{F}\left(\omega ,\;\tau_{D}\right)=\frac{1+\left(5z/8\right)+\left(z^{2}/8\right)}{1+z+\left(z^{2}/2\right)+\left(z^{3}/6\right)+\left(4z^{4}/81\right)+\left(z^{5}/81\right)+\left(z^{6}/648\right)}, \end{array}$$

(13) $$\begin{array}{c} z={\left(2\omega \tau_{D}\right)}^{1/2}, \end{array}$$

(14) $$\begin{array}{c} \tau_{D}=\frac{d^{2}}{D}, \end{array}$$

and *D* is the sum of the diffusion coefficients of the water molecule and of the paramagnetic complex.

The paramagnetic longitudinal and transverse relaxivities correspond to *R*_1*p*_ and *R*_2*p*_, respectively, when the concentration of the paramagnetic ion is 1 mmol/dm^3^.

## 3. Results and Discussion

### 3.1. Metal Content of Pineapple Juice

The amount of copper, iron, and manganese ions in pineapple juice was evaluated through ICP-AES. The resulting concentrations of the three ions were 0.002–0.007 mmol/dm^3^, 0.027–0.031 mmol/dm^3^, and 0.42–0.46 mmol/dm^3^, respectively. The ranges refer to the variability observed for four samples taken from different fruit juice batches. The concentration of Mn^2+^ is more than one order of magnitude larger than that of iron and copper so that the relaxation properties of the juice are largely dictated by the presence of this ion.

### 3.2. ^1^H NMRD Profiles of Pineapple Juice

**Figure 1:**
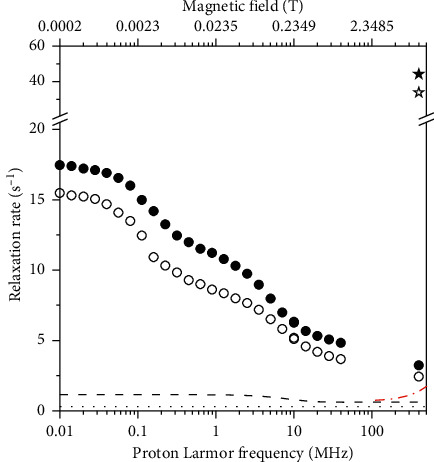
Experimental ^1^H *R*_1_ profiles (circles) and ^1^H *R*_2_ at 400 MHz (stars) of pineapple juice at 25°C (solid symbols) and 37°C (empty symbols). The dotted and dashed lines represent estimates of the diamagnetic contribution and of the latter summed to the *R*_1_ contribution from iron ions at 37°C, respectively; the dotted-dashed red line provides an estimate of the contributions to *R*_2_ from diamagnetic relaxation and from transverse relaxation of iron ions at 37°C.

**Figure 2:**
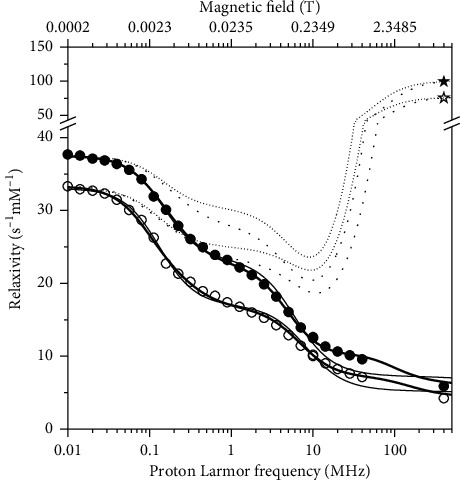
^1^H longitudinal relaxivity profiles (circles) and transverse relaxivity at 400 MHz (stars) of Mn^2+^ ions in pineapple juice at 25°C (solid symbols) and 37°C (empty symbols). The solid and dotted lines represent the best fit profiles of the longitudinal and transverse relaxivities, respectively. The thin lines refer to the fit without inclusion of the order parameter *S*_*LS*_^2^.

The ^1^H longitudinal relaxation profiles measured for pineapple juice at 25 and 37°C are shown in Figure 1. As expected, for water solutions of Mn^2+^ ions, there are two dispersions, one at the lowest frequency corresponding to the Fermi-contact dispersion and the other to the dipolar *ω*_*S*_ dispersion. In order to evaluate the paramagnetic relaxivity due to Mn^2+^, we should estimate the diamagnetic contribution and the contributions from the other paramagnetic ions. The contribution from copper ions should be negligible, due to the low concentration and low relaxivity of this *S* = 1/2 ion [17] and also with respect to the diamagnetic relaxation rate which is expected to be about 0.4 s^−1^. We can figure out the relaxation contribution of iron from the relaxivity of Fe^3+^ aqua ion and its concentration in pineapple juice [25]. Once added to the diamagnetic contribution, we obtain an estimate of the relaxation rates not ascribable to the hyperfine interaction of the water protons with the Mn^2+^ ions, which should amount to the values indicated by the dashed line in Figure 1 (at 37°C). Since these contributions are one order of magnitude smaller than the experimental rates of pineapple juice, we are confident that some inaccuracies in these estimates do not affect the following analysis appreciably. Once subtracted from the measured relaxation rates and normalized to the Mn^2+^ concentration, we obtain the relaxivity of Mn^2+^ ions in pineapple juice, as shown in Figure 2.

The transverse relaxation rates of pineapple juice at 400 MHz and 25 and 37°C were also measured (shown as star symbols in Figure 1). Again, the rates are more than one order of magnitude larger than the expected contribution from iron ions [25] and diamagnetic terms (dotted-dashed line in Figure 1). The calculated *R*_2_ relaxivity of Mn^2+^ ions in pineapple juice results as large as 100 and 75 s^−1^·mM^−1^ at 25 and 37°C, respectively (star symbols in Figure 2).

**Figure 3:**
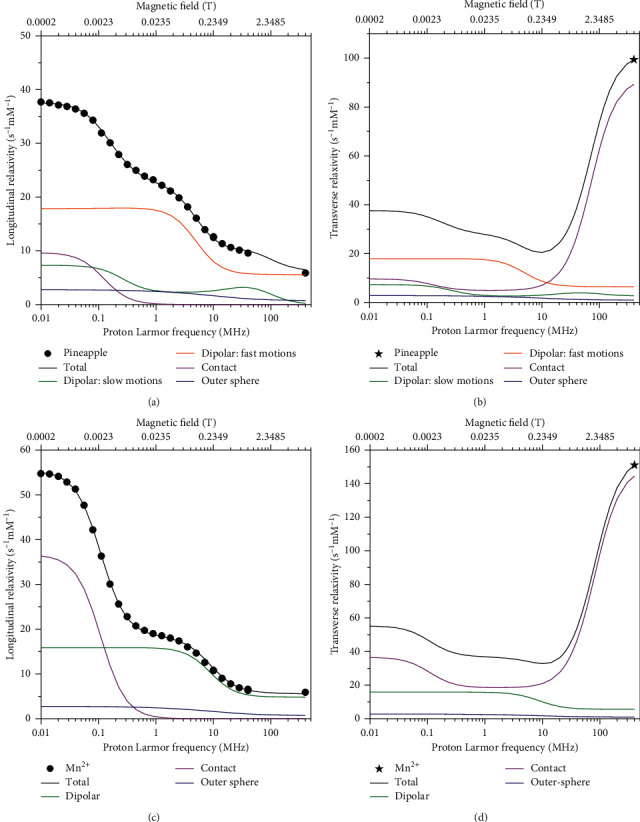
^1^H longitudinal relaxivity (a, c) and transverse relaxivity (b, d) at 25°C and their dipolar, Fermi-contact, and outer sphere contributions in pineapple juice (a, b) and in Mn^2+^ solution (c, d).

The longitudinal and transverse relaxivity data of Mn^2+^ ions in pineapple juice were fit to the model described by equations (1)–(13). In the fit, the Mn-water proton distances *r* were fixed to 2.85 Å, the distance of closest approach *d* to 3.6 Å, and the diffusion coefficients at 25 and 37°C to 3.0 × 10^−9^ and 3.9 × 10^−9^ m^2^/s, respectively. As a first step, the number of coordinated water molecules *q*, the reorientation correlation time *τ*_*r*_, the water proton lifetime *τ*_*M*_, the electronic parameters Δ_*t*_ and *τ*_*v*_, and the constant of contact coupling (*A*^FC^/*h*)were left free to be adjusted, with *S*_*LS*_^2^=1. The resulting best fit is of low quality, as shown in Figure 2 as thin lines (we checked that the quality of the fit does not improve even if the parameters indicated as fixed are left free to adjust). Therefore, a squared order parameter *S*_*LS*_^2^ was introduced to account for the presence of contributions arising from two different reorientation correlation times, namely, *τ*_*r*_ and *τ*_*ι*_, where *τ*_*ι*_ is much smaller than *τ*_*r*_. Using this model-free approach, it is thus possible to consider the relaxation contributions from water molecules experiencing overall and local dynamics occurring on quite different time scales. In this way, the fit was very good (thick lines in Figure 2). The best fit values of the parameters are reported in Table 1. The value of Δ_*t*_ is completely covariant with the values of *τ*_*v*_ and *τ*_*M*_, and it can thus be fixed (in Table 1, it was fixed to the value obtained from the analysis of the profiles collected in the presence of alginate). The contributions from the dipolar interaction modulated by slow mobility and by fast mobility, from the Fermi-contact interaction as well as from outer sphere relaxation, are shown in Figure 3. The figure shows that the largest contribution to longitudinal relaxation originates from the metal-proton dipole-dipole interactions modulated by reorientation motions with correlation times of about 40–50 ps. The proton lifetime is relatively small, of the order of tens of nanoseconds. A Fermi-contact contribution is present with a contact coupling constant of 0.55 MHz. Fermi-contact relaxation is responsible of the first inflection in the longitudinal relaxation profile and is basically responsible (together with the water proton lifetime and field-dependent electron relaxation time) for the very high transverse relaxation at 400 MHz. The need for minor contributions (2%) from dynamics in the nanosecond regime, required for achieving a good fit of the data, suggests that the manganese ions interact with some macromolecules present in the juice. Consistently, the occurrence of these interactions decreases the number of water molecules coordinated to Mn^2+^ (*q*) from 6 to 4.

### 3.3. ^1^H NMRD Profiles of Mn^2+^ Solution

**Figure 4:**
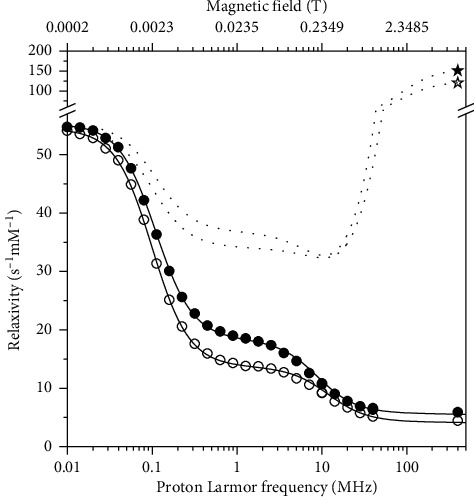
^1^H longitudinal relaxivity profiles (circles) and transverse relaxivity at 400 MHz (stars) of Mn^2+^ solutions (pH 3.6) at 25°C (solid symbols) and 37°C (empty symbols). The solid and dotted lines represent the best fit profiles of the longitudinal and transverse relaxivities, respectively.

In order to check whether the relaxivity profiles of pineapple juice is only determined by the relaxivity of the manganese ions in water solution, or whether there are effects from the other components of the juice, the relaxivity profiles of a Mn^2+^ solution at the same pH (3.6) and salt concentrations of pineapple juice were obtained (Figure 4). The low-field relaxivity in this case is larger, and the Fermi-contact (low field) dispersion is higher in Mn^2+^ solution than in pineapple juice, whereas the high-field relaxivity is smaller. This suggests that the contact coupling constant in Mn^2+^ solution is larger than in pineapple juice. The position of the dipolar (high field) dispersion is at larger frequency in Mn^2+^ solution than in pineapple juice, thus indicating a shorter reorientation correlation time for dipolar relaxation. The *R*_2_ values of Mn^2+^ at 400 MHz are somewhat larger than those of pineapple juice. In this case, the fit obtained assuming six water molecules regularly coordinated to the manganese ion was excellent (solid lines in Figure 4). The best fit parameters are reported in Table 2 and are in good agreement with previous results obtained for Mn^2+^ aqua ions [26]. The relative contributions of Fermi-contact and dipolar inner sphere and outer sphere relaxation are shown in Figure 3. Different from pineapple juice, the complex is free to reorient with correlation times of 20–30 ps, as expected for an aqua ion. The contact coupling constant is 0.82 MHz. This value is significantly larger in Mn^2+^ solution than in pineapple juice likely because in the latter, water coordination is somewhat hampered by macromolecular interactions. Clearly, Fermi-contact relaxation provides the major contribution to the longitudinal relaxivity at low fields, whereas dipolar relaxation provides the major contribution at high fields. However, the Fermi-contact interaction represents by far the largest source for transverse relaxation at 400 MHz. The lifetime of the coordinated water protons is similar to that of pineapple juice.

### 3.4. ^1^H NMRD Profiles of Pineapple Juice in the Presence of Alginate

As a general strategy to increase the relaxation rates at high fields, the molecular dynamics of the paramagnetic complex can be slowed down by promoting transient interactions between the paramagnetic complex and macromolecules [1, 27, 28]. The relaxivity of Gd^3+^ contrast agents, for instance, has a peak at fields of about 1 T when the Gd^3+^ complex binds proteins [29–31], is coordinated to nanoparticles [32–37], or is entrapped into hydrogels [38, 39]. In this case, we aim at introducing a food stuff containing large macromolecules with which the Mn^2+^ ion can transiently interact or which may cause its confinement in a restricted environment, in such a way as to reduce its mobility. Alginate, a polysaccharide which is a commercially available natural food, was used for this purpose. Since sodium alginate increases dramatically the viscosity of the solution, the relaxometric analysis can provide crucial data to optimize the amount of polysaccharide in order to obtain a high contrast and suitable rheological properties. After a preliminary screening of sodium alginate concentrations, solutions at 5% and 15% in weight were investigated to determine the physicochemical parameters influencing the relaxation rates of these hydrogels. While solutions at 5% in weight of sodium alginate are still viscous liquids, solutions at 15% appear to be thick pastes.

**Figure 5:**
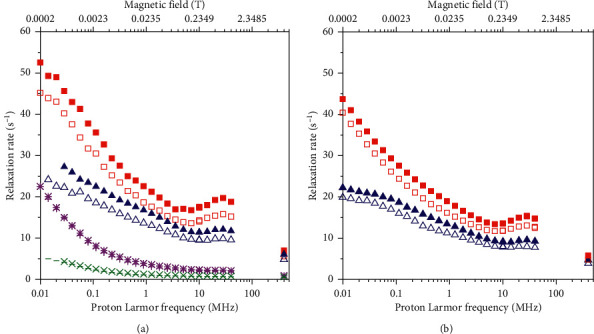
(a) Experimental ^1^H R1 profiles of pineapple juice with alginate (red squares: 15%, and blue triangles: 5% w/w) at 25°C (solid symbols) and 37°C (empty symbols), and of alginate solutions (pink symbols: 15%, and green symbols: 5% w/w; ∗ and ×: 25 °C, + and ‐: 37 °C). (b). Experimental 1H R1 profiles of the Mn^2+^ solutions with alginate (red squares: 15%, and blue triangles: 5% w/w) at 25°C (solid symbols) and 37°C (empty symbols).

The diamagnetic relaxation rates of alginate solutions at 5% and 15% in weight are shown in Figure 5(a). These solutions were prepared in sodium acetate, in order to have the same pH of pineapple juice and by addition of 4 mmol/dm^3^ (150 mg/L) concentration of Ca^2+^ ions, as present in the commercial juice, which promotes the formation of a hydrogel [40]. The large increase in the relaxation rates, which can be observed by decreasing the magnetic field, actually indicates cross-linking of alginate polymers [41–43]. Alginate in the same concentration was also added to pineapple juice, and the relaxation profiles were measured (Figure 5(a)). At all fields, the relaxation rates in this case are sizably larger than those measured for the pristine pineapple juice (Figure 1); the steep decrease in relaxation at low fields parallels the decrease seen for the alginate sample and is thus ascribed to the diamagnetic contribution, whereas the relaxivity peak observed at about 1 T originates from the restricted mobility of the paramagnetic ion and the field dependence of the electron relaxation rates.

**Figure 6:**
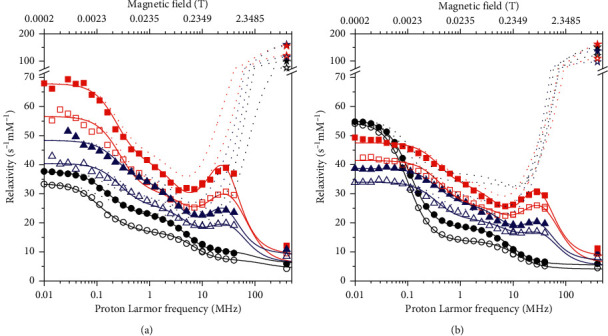
(a) ^1^H longitudinal relaxivity profiles and transverse relaxivity at 400 MHz (stars) of Mn^2+^ ions in pineapple juice without (black symbols) and with alginate (5%: blue symbols; 15%: red symbols) at 25°C (solid symbols) and 37°C (empty symbols). (b) ^1^H longitudinal relaxivity profiles and transverse relaxivity at 400 MHz (stars) of Mn^2+^ solutions without (black symbols) and with alginate (5%: blue symbols; 15%: red symbols) at 25°C (solid symbols) and 37°C (empty symbols). The solid and dotted lines represent the best fit profiles of the longitudinal and transverse relaxivities, respectively.

**Figure 7:**
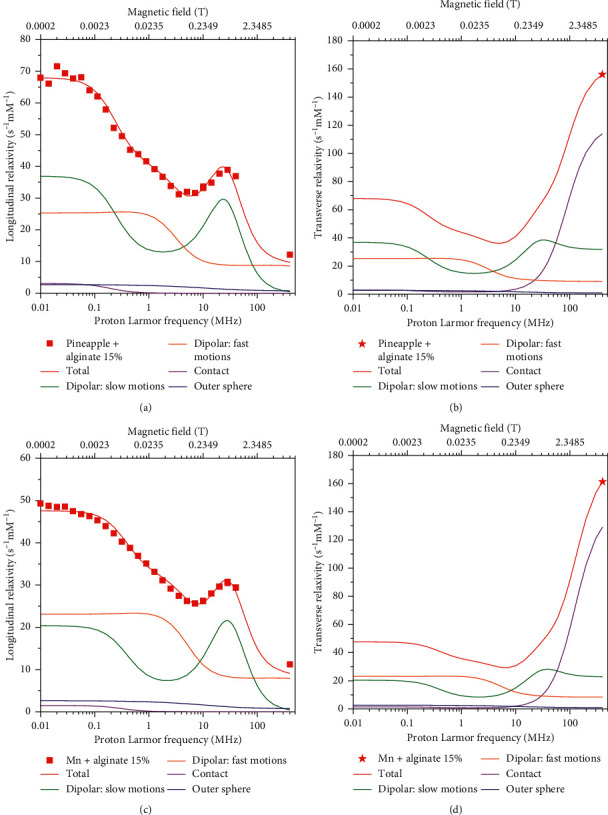
^1^H longitudinal relaxivity (a, c) and transverse relaxivity (b, d) at 25 °C and their dipolar, Fermi-contact, and outer-sphere contributions in the pineapple juice (a, b) and in the Mn^2+^ solution (c, d) upon addition of 15% w/w alginate.

Figure 6 shows the relaxivity data for the samples of pineapple juice with addition of alginate, obtained after subtraction of the diamagnetic relaxation rates of alginate and of the paramagnetic contribution from iron ions, and subsequent normalization to 1 mmol/dm^3^ Mn^2+^ concentration. The paramagnetic contribution from iron ions is assumed similar to that considered in the absence of alginate because of the polymer chelation of the Fe(III) ions at this pH [44], with subsequent increase in the reorientation time and decrease in the number of bound water molecules. The relaxivity profiles of pristine pineapple juice are also reported in Figure 6 for an easier comparison, as well as the transverse relaxivity of all samples at 400 MHz. The data were fit using equations (1)–(13), including an order parameter, and the best fit parameters are shown in Table 1. The contributions from the dipolar interaction modulated by slow mobility and fast mobility, from the Fermi-contact interaction as well as from outer sphere relaxation, are shown in Figures 7(a) and 7(b) for the sample with 15% alginate at 25°C.

The fit indicates a similar number of water molecules coordinated to the Mn^2+^ ions upon alginate addition (the small increase in the best fit value of *q* may just reflect larger outer and/or second sphere contributions arising from the higher viscosity of the system, which may decrease the microscopic diffusion constant and slower the lifetime of second sphere water molecules). The analysis indicates a decrease in the value of the contact coupling constant in the presence of alginate and a sizable increase of the lifetime of the coordinated water molecules and of the value of *S*_*LS*_^2^.

### 3.5. ^1^H NMRD Profiles of Mn2+ Solution in the Presence of Alginate

Finally, the relaxation profiles were measured for Mn^2+^ solution in the presence of alginate (Figure 5(b)). The shapes of the profiles are very similar to those observed for pineapple juice although the rates are in this case somewhat smaller. The corresponding relaxivity profiles are shown in Figure 6(b). Like in the previous cases, the data were fit using equations (1)–(13), and the best fit parameters are reported in Table 2. Figures 7(c) and 7(d) show the contributions from the dipolar interaction modulated by slow mobility and by fast mobility, from the Fermi-contact interaction, as well as from outer sphere relaxation for the sample with 15% alginate at 25°C. Also, in this case, there is a reduction in the value of the contact coupling constant with respect to the pristine Mn^2+^ solution and a large increase in the lifetime of the coordinated water molecules. As for pineapple juice, there are contributions from dipolar relaxation with reorientation times of the order of nanoseconds, which originate the relaxation peak at about 1 T.

A lower order parameter is obtained for Mn^2+^ solution than for pineapple juice (in the presence of alginate), and this causes the observed smaller relaxivity measured for the Mn^2+^ solutions, despite that the opposite trend was measured in the absence of alginate. Overall, the analysis indicates a lower restriction in the mobility of the Mn^2+^ complex caused by alginate in this solution with respect to pineapple juice.

### 3.6. Contribution of Hydrogel to the Negative Contrast

**Figure 8:**
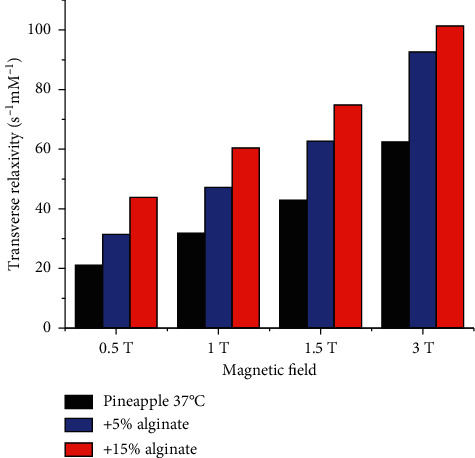
Transverse relaxivity of pineapple juice without and with alginate at 37°C.

The profiles of the transverse relaxivity in the range 0.01–400 MHz, generated from the fit of the experimental data, allow for a detailed evaluation of the contrast enhancement of pineapple juice in the hydrogel formulations, at the magnetic fields used in clinical MRI (Figure 8). It is interesting to observe that the hydrogel formulations exhibit sizable larger transverse relaxivities than the pristine pineapple juice, proportional to the alginate concentration. On the contrary, the transverse relaxivities of Mn^2+^ solutions are almost unaffected by the presence of alginate (Figure 6(b)).

## 4. Conclusions

The acquired ^1^H NMRD profiles confirm that the relaxation rates measured for pineapple juice are mostly determined by the large relaxivity of the Mn^2+^ ions, as expected. However, the analysis of the profiles indicates that the number of water molecules coordinated to the Mn^2+^ ions is about 4, instead of 6, as in the aqua ions, the Fermi-contact coupling constant is smaller than for the Mn^2+^ aqua ion, and the tumbling time of the Mn^2+^ complex has components in the nanosecond time scale, likely due to a fraction of metal ions with mobility restricted by interactions with macromolecules present in the juice. The presence of alginate can further slower the tumbling time, and thus the relaxation rates, the effect being larger for pineapple juice than for Mn^2+^ solution. This makes the values of *R*_1_ at 0.94 T higher for pineapple juice than for Mn^2+^ solution with the same ion concentration, both in the absence and in the presence of alginate (Table 3). At 9.4 T, the longitudinal relaxation rates are somewhat smaller than at 0.94 T, especially for the samples in the presence of alginate. However, the high transverse relaxation rates of pineapple juice are largely increased upon addition of alginate (of about a factor 2). The *R*_2_ measured at 9.4 T for Mn^2+^ solution is somewhat larger than for pineapple juice due to the larger contact coupling constant, but in this case, the increase in the rate is minor upon addition of alginate, due to the lifetime of the water molecules coordinated to the metal ions which is limiting the increase in the transverse relaxation.

Collectively, these data provide new hints for the possible use of a hydrogel prepared from pineapple juice, as negative oral contrast agent in magnetic resonance imaging. It should be noted that the profiles of the transverse relaxivity as a function of the applied magnetic field strength show that the increased viscosity can improve significantly the negative contrast at physiological temperature and MRI fields of 0.5–1 T (and at lower magnetic fields, see Figures 6 and 8). Conversely, at any magnetic field values, the increase of the viscosity does not enhance significantly the transverse relaxivity of solutions obtained by dissolving Mn^2+^ salts. These results suggest that the macromolecular components that are present in pineapple juice play a pivotal role in the observed relaxivity enhancement, most likely by immobilizing the Mn^2+^ ions of the solution to a larger extent.

## Figures and Tables

**Table 1:** Best fit parameters for pineapple juice without and with addition of alginate 5% or 15% w/w.

	Pineapple				+5% alginate		+15% alginate		
	25°C	37°C	25°C	37°C	25°C	37°C	25°C	37°C	
*r*(^*∗*^)	2.85								Å
*q*	5.3 ± 0.3		4.0 ± 0.1		4.5 ± 0.3		4.3 ± 0.4		
Δ_*t*_	0.015 ± 0.001								cm^−1^
*τ*_*r*_	42 ± 3	30 ± 2	1700 ± 300	1000 ± 200	4700 ± 1100	2900 ± 800	4700 ± 1200	2700 ± 800	ps
*τ*_*v*_	11 ± 1	9 ± 1	9 ± 1	7 ± 1	16 ± 1	16 ± 1	16 ± 3	16 ± 3	ps
*τ*_*M*_	22 ± 2	16 ± 2	39 ± 3	29 ± 2	200 ± 90	91 ± 60	140 ± 80	92 ± 60	ns
*S*_*LS*_^2^	—		0.021 ± 0.003		0.05 ± 0.01		0.10 ± 0.01		
*τ*_*l*_	—	—	51 ± 2	36 ± 2	74 ± 8	49 ± 6	83 ± 15	54 ± 11	ps
*A* ^FC^/*h*	0.63 ± 0.02		0.55 ± 0.02		0.37 ± 0.02		0.38 ± 0.05		MHz

Outer sphere relaxation was also included with *d* = 3.6 Å and *D* = 3.0·10^−5^ and 3.9·10^−5^cm^2^/s at 25°C and 37°C, respectively. (^*∗*^) fixed.

**Table 2:** Best fit parameters for Mn^2+^ solution without and with addition of alginate 5% or 15% w/w.

	Mn^2+^ solution		+5% alginate		+15% alginate		
	25°C	37°C	25°C	37°C	25°C	37°C	
*r*(^*∗*^)	2.85						Å
*q*	6						
Δ_*t*_	0.018 ± 0.001						cm^−1^
*τ*_*r*_	28 ± 1	20 ± 1	4200 ± 1400	3100 ± 1200	3800 ± 600	2700 ± 500	ps
*τ*_*v*_	5.3 ± 0.1	4.5 ± 0.1	17 ± 2	16 ± 2	18 ± 1	18 ± 1	Ps
*τ*_*M*_	18 ± 1	14 ± 1	44 ± 16	29 ± 11	220 ± 100	100 ± 70	ns
*S*_*LS*_^2^	—		0.03 ± 0.01		0.06 ± 0.01		
*τ*_*l*_	—	—	39 ± 1	29 ± 1	51 ± 3	36 ± 2	ps
*A* ^FC^/*h*	0.82 ± 0.01		0.48 ± 0.08		0.29 ± 0.02		MHz

Outer sphere relaxation was also included with *d* = 3.6 Å and *D* = 3.0·10^−5^ and 3.9·10^−5^cm^2^/s at 25°C and 37°C, respectively. (^*∗*^) fixed.

**Table 3:** Relaxation rates (s^−1^) at 37°C measured for pineapple juice and Mn^2+^ solutions without and with alginate.

	*R*_1_ at 0.94 T	*R*_1_ at 9.4 T	*R*_2_ at 9.4 T
Pineapple juice	3.7	2.4	33.9
Pineapple juice + 5% alginate	9.6	4.8	58.4
Pineapple juice + 15% alginate	15.2	5.6	73.7
Mn^2+^ solution 0.43 mmol/dm^3^, pH 3.6	2.5	2.2	52.1
Mn^2+^ solution 0.43 mmol/dm^3^ + 5% alginate	7.7	3.9	49.5
Mn^2+^ solution 0.43 mmol/dm^3^ + 15% alginate	12.6	4.8	68.0

## Data Availability

The data used to support the findings of this study are available upon request to Giacomo Parigi and Marco Fragai.
